# Establishment and validation of a predictive nomogram model for non-small cell lung cancer patients with chronic hepatitis B viral infection

**DOI:** 10.1186/s12967-018-1496-5

**Published:** 2018-05-04

**Authors:** Shulin Chen, Yanzhen Lai, Zhengqiang He, Jianpei Li, Xia He, Rui Shen, Qiuying Ding, Hao Chen, Songguo Peng, Wanli Liu

**Affiliations:** 1 State Key Laboratory of Oncology in South China, Collaborative Innovation Center for Cancer Medicine, Sun Yat-sen University Cancer Center, 651 Dongfeng Road East, Guangzhou, 510060 China; 2 Guangdong Provincial Key Laboratory of Malignant Tumor Epigenetics and Gene Regulation, Department of Clinical Laboratory Medicine, Sun Yat-Sen Memorial Hospital, Sun Yat-Sen University, Guangzhou, 510020 China

**Keywords:** Hepatitis B viral, Liver function, Nomogram, Non-small cell lung cancer, Prognosis

## Abstract

**Background:**

This study aimed to establish an effective predictive nomogram for non-small cell lung cancer (NSCLC) patients with chronic hepatitis B viral (HBV) infection.

**Methods:**

The nomogram was based on a retrospective study of 230 NSCLC patients with chronic HBV infection. The predictive accuracy and discriminative ability of the nomogram were determined by a concordance index (C-index), calibration plot and decision curve analysis and were compared with the current tumor, node, and metastasis (TNM) staging system.

**Results:**

Independent factors derived from Kaplan–Meier analysis of the primary cohort to predict overall survival (OS) were all assembled into a Cox proportional hazards regression model to build the nomogram model. The final model included age, tumor size, TNM stage, treatment, apolipoprotein A-I, apolipoprotein B, glutamyl transpeptidase and lactate dehydrogenase. The calibration curve for the probability of OS showed that the nomogram-based predictions were in good agreement with the actual observations. The C-index of the model for predicting OS had a superior discrimination power compared with the TNM staging system [0.780 (95% CI 0.733–0.827) vs. 0.693 (95% CI 0.640–0.746), *P* < 0.01], and the decision curve analyses showed that the nomogram model had a higher overall net benefit than did the TNM stage. Based on the total prognostic scores (TPS) of the nomogram, we further subdivided the study cohort into three groups: low risk (TPS ≤ 13.5), intermediate risk (13.5 < TPS ≤ 20.0) and high risk (TPS > 20.0).

**Conclusion:**

The proposed nomogram model resulted in more accurate prognostic prediction for NSCLC patients with chronic HBV infection.

## Background

Lung cancer remains the most common cause of cancer deaths worldwide. The recent literature reported that 5-year survival rate of lung cancer was 18% [1]. More than 80% of total lung cancer cases are non-small cell lung cancer (NSCLC) [2]. The low survival rates of NSCLC patients are affected by many factors, including poor early diagnosis, tumor recurrence, and distant metastasis.

Chronic hepatitis B viral (HBV) infection is also a serious global public problem. Two billion people are estimated to be infected with HBV worldwide [3]. In particular, China has a high prevalence of HBV infections, and HBV patients in China account for approximately 38% of all patients worldwide [4]. A national epidemiology survey announced in April 2008 by the Ministry of Health showed that 93 million people in China had been infected with HBV.

Numerous studies have reported that many prognostic factors are correlated with the prognosis of NSCLC patients. The prognostic factors include liver function indicators (LFIs), such as aspartate aminotransferase (AST) [5], lactate dehydrogenase (LDH) [6], and alkaline phosphatase (ALP) [7], among others.

The risk of liver injury may increase when HBV infects patients. Most studies suggest that liver injury in viral hepatitis is not caused by the direct cytopathic effects of viruses but by the host immune response to viral proteins expressed by infected hepatocytes [8], which causes liver dysfunction. Research has shown that chronic HBV infection is an independent prognostic factor in patients with nasopharyngeal carcinoma [9], pancreatic cancer [10] and NSCLC [11]. These results suggest that NSCLC patients with HBV infection should be distinguished from those without HBV infection because they have different clinicopathological characteristics, prognostic factors, and outcomes after treatment, which require a distinct prognostic, predictive model.

Nomogram is currently widely applied as graphical representations of complex mathematical formulas. They can integrate essential factors to build a statistical prognostic model for estimating prognosis in the outcomes of many cancers [12, 13]. Furthermore, nomogram has been shown to make more precise predictions than do the traditional staging systems used in many cancers [14, 15]. However, no study has established a prognostic nomogram for NSCLC patients with HBV. Therefore, our study aimed to develop a practical clinical tool by combining clinicopathologic factors and markers of liver function tests. We also tested whether the nomogram model provides a more accurate prediction of patient survival than does the 7th edition of the American Joint Committee on Cancer (AJCC) TNM Staging.

## Methods

### Patients and study design

A retrospective observational study was performed including a total of 230 NSCLC patients with chronic HBV infection, and the patients first visited Sun Yat-sen University Cancer Center (Guangzhou, China) between January 2008 and December 2010. The inclusion criteria were as follows: (1) patients with a confirmed pathological diagnosis of NSCLC; (2) patients who were positive for the hepatitis B surface antigen (HBsAg), excluding acute hepatitis; (3) patients without co-infection of other types of hepatitis viruses; (4) patients with complete clinical data; (5) patients without secondary carcinomas as assessed by clinical history, computed tomography (CT), ultrasonographic examination and routine laboratory tests; and (6) patients without liver fibrosis, steatosis, and cirrhosis as detected by CT or ultrasonographic examination.

All the samples were collected at the time of diagnosis before any treatment. We obtained the patients’ clinicopathologic parameters including gender, age, family history, smoking history, body mass index (BMI), pathologic TNM stage [16], tumor size, and treatment history. LFIs including alanine transaminase (ALT), aspartate aminotransferase (AST), the AST-to-ALT ratio (SLR), apolipoprotein A-I (APOAI), apolipoprotein B (APOB), alkaline phosphatase (ALP), albumin (ALB), glutamyl transpeptidase (GGT), lactate dehydrogenase (LDH), total bilirubin (TBIL) and direct bilirubin (DBIL) and HBV infection markers including HbsAg, hepatitis B surface antibody (HbsAb), hepatitis B e antigen (HbeAg), hepatitis B e antibody (HbeAb) and hepatitis B core antibody (HbcAb) were recorded.

We randomly divided the patients into a primary cohort and a validation cohort. Computer-generated random numbers were used to assign 141 of the patients to the primary cohort and 89 patients to the validation cohort. The overall survival (OS) of the patients was recorded based on a follow-up clinical visit or a telephone call. The OS was calculated from the time of initial diagnosis until the time of death from any cause, or until the last follow-up. All the patients were followed up until death or January 2016, if still alive. The authenticity of this article has been validated by uploading the key raw data onto the Research Data Deposit public platform (http://www.researchdata.org.cn), with the approval RDD Number as RDDA2018000554.

### Laboratory measurements

ALT, AST, APOAI, APOB, ALP, ALB, GGT, LDH, TBIL, and DBIL were measured using a Hitachi 7600 Automatic Analyzer (Tokyo, Japan). HBsAg, HbsAb, HbeAg, HbeAB, and HbcAb were detected by enzyme-linked immunosorbent assay (ELISA) technology. The values of all the variables tested a few days before pretreatment were recorded. The SLR was defined as the serum AST level divided by the serum ALT level. The coefficient of variation for these methods over the range of measurements was < 5% as established by routine quality control procedures.

### Statistical analysis

Categorical variables were classified based on clinical findings, and continuous variables were transformed into categorical variables based on cutoff points, which were determined by the minimum P value from log-rank ×2 statistics using the X-tile program [17]. Survival curves were depicted using the Kaplan–Meier method and compared with a log-rank test stratified according to the prognostic factors. The P values of variables less than or equal to 0.05 in the univariate analyses were incorporated into the Cox’s proportional hazards regression. A predictive nomogram model was built based on the Cox model parameter estimates in the primary cohort, and the selection of the final prediction model was performed with a backward step-down selection process using the Akaike information criterion [18]. The accuracy of the nomogram model was estimated by the Harrell’s C-index (C-index). The value of the C-index ranges from 0.5 to 1.0, with 0.5 indicating random chance and 1.0 indicating a perfect ability to correctly discriminate the outcome with the model. Validation was performed using a bootstrap method to quantify our modeling strategy. Finally, a calibration curve of the nomogram model for the 1-, 3-, and 5-year OS and decision curve analyses [19] was plotted to assess the predictive value of the model. The nomogram model was divided into three groups (low-risk prognosis, intermediate-risk prognosis, and high-risk prognosis) based on the total prognostic scores (TPS) in the primary cohort and validation cohort. Correlation analysis was adopted using Pearson’s correlation. All the statistical analyses and graphics were performed using the SPSS 20.0 statistical package (SPSS Inc., Chicago, IL, USA) and R version 3.3.2 (http://www.r-project.org/). P values less than 0.05 were considered statistically significant.

## Results

### Patient characteristics

The study enrolled a total of 230 patients. We randomly divided the patients into a primary cohort and a validation cohort. The patients’ demographic and clinical characteristics are listed in Table 1. There were 141 patients in the primary cohort, comprising 72 male patients (51.1%) and 69 female patients (48.9%), and the age of the patients ranged from 27 to 82 years. The median follow-up time was 33 months. The validation cohort included 50 male patients (56.2%) and 39 female patients (43.8%), and the age of the patients ranged from 33 to 79 years. The median follow-up time was 35 months. The 5-year OS rates for the primary cohort and the validation cohort were 33.3 and 33.7%, respectively.

**Table 1:** Patient demographics and clinical characteristics

	Primary cohort	Validation cohort
	n = (141)	n = (89)
Characteristic	No. (%)	No. (%)
Gender		
Male	72 (51.1%)	50 (56.2%)
Female	69 (48.9%)	39 (43.8%)
Age (years)		
≤ 42	19 (13.5%)	11 (12.4%)
> 42	122 (86.5%)	78 (87.6%)
Family history		
Yes	36 (25.5%)	18 (20.2%)
No	105 (74.5%)	71 (79.8%)
Smoking behaviour		
Yes	84 (59.6%)	50 (56.2%)
No	57 (40.4%)	39 (43.8%)
BMI		
≥ 25	30 (21.3%)	14 (15.7%)
18.5 ≤ BMI < 25	103 (73.0%)	65 (73.0%)
< 18.5	8 (5.7%)	10 (11.3%)
TNM stage^a^		
I	31 (22.0%)	23 (25.8%)
II	17 (12.1%)	6 (6.7%)
III	53 (37.6%)	34 (38.2%)
IV	40 (28.3%)	26 (29.3%)
Size (cm)^b^		
≤ 6.3	122 (86.5%)	70 (78.7%)
> 6.3	19 (13.5%)	19 (21.3%)
Treatment		
Surgery	37 (26.2%)	21 (23.6%)
Radiotherapy/chemotherapy	47 (33.3%)	31 (34.8%)
Surgery and radiotherapy/chemotherapy	49 (34.8%)	25 (28.1%)
Other	8 (5.7%)	12 (13.5%)
ALT (U/L)		
≤ 12.5	15 (10.6%)	11 (12.4%)
> 12.5	126 (89.4%)	78 (87.6%)
AST (U/L)		
≤ 22.0	73 (51.8%)	45 (50.1%)
> 22.0	68 (48.2%)	44 (49.9%)
SLR		
≤ 1.24	102 (72.3%)	61 (68.5%)
> 1.24	39 (27.7%)	28 (31.5%)
APOAI (g/L)		
≤ 1.17	56 (39.7%)	39 (43.8%)
> 1.17	85 (60.3%)	50 (56.2%)
APOB (g/L)		
≤ 0.99	92 (65.2%)	50 (56.2%)
> 0.99	49 (34.8%)	39 (43.8%)
ALP (U/L)		
≤ 82.9	96 (68.1%)	51 (57.3%)
> 82.9	45 (31.9%)	38 (42.7%)
ALB (g/L)		
≤ 40.1	108 (76.6%)	62 (69.7%)
> 40.1	33 (23.4%)	27 (30.3%)
GGT (U/L)		
≤ 44.2	117 (83.0%)	72 (80.9%)
> 44.2	24 (17.0%)	17 (19.1%)
LDH (U/L)		
≤ 245.6	125 (88.7%)	77 (86.5%)
> 245.6	16 (11.2%)	12 (13.5%)
TBIL (μmol/L)		
≤ 13.6	113 (80.1%)	61 (68.5%)
> 13.6	28 (19.9%)	28 (31.5%)
DBIL (μmol/L)		
≤ 3.3	67 (47.5%)	38 (42.7%)
> 3.3	74 (52.5%)	51 (57.2%)

*BMI* body mass index, *TNM* pathological tumour node metastasis stage, *ALT* alanine transaminase, *AST* aspartate aminotransferase, *SLR* AST-to-ALT ratio, *APOAI* apolipoprotein AI, *APOB* apolipoprotein B, *ALP* alkaline phosphatase, *ALB* albumin, *GGT* glutamyl transpeptidase, *LDH* lactic dehydrogenase, *TBIL* total bilirubin, *DBIL* direct bilirubin

^a^TNM stage was classified according to the AJCC 7th TNM staging system

^b^The tumor maximum diameter

### Univariate analysis of the OS in the primary cohort

In the univariate analysis (Table 2), Kaplan–Meier survival analysis and log-rank tests were performed to predict patients’ OS. Univariate analysis indicated that age (*P* < 0.001), TNM stage (*P* < 0.001), tumor size (*P* = 0.001), treatment (*P* < 0.001), SLR (*P* = 0.044), APOAI (*P* < 0.001), APOB (*P* = 0.024), ALP (*P* = 0.018), GGT (*P* = 0.003) and LDH (*P* < 0.001) were significantly associated with OS in NSCLC patients with HBV infection.

**Table 2:** Univariate analysis of OS in the primary cohorts

Variable	Univariate analysis	
	HR (95% CI)	*P*
Gender		
Male	Reference	
Female	1.088 (0.678–1.746)	0.726
Age (years)		
≤ 42	Reference	
> 42	0.316 (0.171–0.587)	< 0.001
Family history		
Yes	Reference	
No	0.877 (0.512–1.501)	0.631
Smoking behaviour		
Yes	Reference	
No	0.965 (0.595–1.564)	0.884
BMI		
≥ 25	Reference	
18.5 ≤ BMI < 25	1.419 (0.757–2.660)	0.275
< 18.5	1.489 (0.480–4.618)	0.491
TNM stage		
I	Reference	
II	1.058 (0.318–3.517)	0.927
III	3.256 (1.477–7.181)	0.003
IV	6.575 (2.939–14.711)	< 0.001
Size (cm)^d^		
≤ 6.3	Reference	
> 6.3	2.769 (1.503–5.101)	0.001
Treatment		
Surgery	Reference	
Radiotherapy/chemotherapy	3.737 (1.891–7.387)	< 0.001
Surgery and radiotherapy/chemotherapy	1.049 (0.517–2.126)	0.895
Other	6.845 (2.630–17.818)	< 0.001
ALT (U/L)		
≤ 12.5	Reference	
> 12.5	0.630 (0.311–1.278)	0.200
AST (U/L)		
≤ 22.0	Reference	
> 22.0	1.366 (0.849–2.197)	0.198
SLR		
≤ 1.24	Reference	
> 1.24	1.673 (1.015–2.759)	0.044
APOAI (g/L)		
≤ 1.17	Reference	
> 1.17	0.393 (0.244–0.633)	< 0.001
APOB (g/L)		
≤ 0.99	Reference	
> 0.99	0.533 (0.308–0.922)	0.024
ALP (U/L)		
≤ 82.9	Reference	
> 82.9	1.800 (1.106–2.929)	0.018
ALB (g/L)		
≤ 40.1	Reference	
> 40.1	0.693 (0.385–1.246)	0.220
GGT (U/L)		
≤ 44.2	Reference	
> 44.2	2.371 (1.348–4.169)	0.003
LDH (U/L)		
≤ 245.6	Reference	
> 245.6	3.423 (1.847–6.345)	< 0.001
TBIL (μmol/L)		
≤ 13.6	Reference	
> 13.6	0.541 (0.276–1.059)	0.073
DBIL (μmol/L)		
≤ 3.3	Reference	
> 3.3	1.583 (0.974–2.572)	0.064

*BMI* body mass index, *TNM* pathological tumour node metastasis stage, *ALT* alanine transaminase, *AST* aspartate aminotransferase, *SLR* AST-to-ALT ratio, *APOAI* apolipoprotein AI, *APOB* apolipoprotein B, *ALP* alkaline phosphatase, *ALB* albumin, *GGT* glutamyl transpeptidase, *LDH* lactic dehydrogenase, *TBIL* total bilirubin, *DBIL* direct bilirubin

### Prognostic nomogram model for OS

**Fig. 1:**
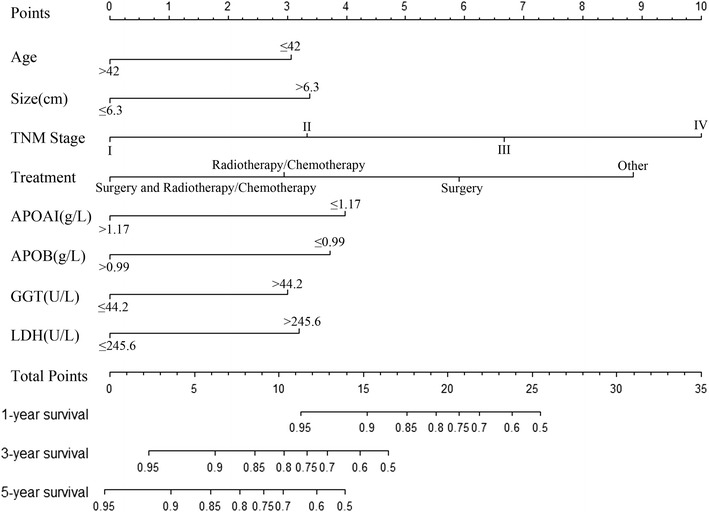
Nomogram model predicting the 1-, 3- and 5-year OS in NSCLC patients with chronic HBV infection. The nomogram was used summing the points identified on the points scale for each variable. The total points projected on the bottom scales indicate the probability of 1-, 3- and 5-year survival

The variables age, TNM stage, tumor size, treatment, SLR, APOAI, APOB, ALP, GGT and LDH were identified as predictors of OS in the univariate analysis and were entered into the Cox’s proportional hazards regression. The selection of the final model was performed using a backward step-down selection process with the Akaike information criterion (AIC). We selected the optimal model based on the smallest AIC. The factors in our final model included age, TNM stage, tumor size, treatment, APOAI, APOB, GGT and LDH, and the AIC of the final model was 559.41. The nomogram model was constructed based on the selected factors, and Fig. 1 shows the nomogram model predicting the 1-, 3- and 5-year OS in the primary cohort. Patients with higher scores corresponded to worse prognoses.

### Internal and external validation of the nomogram model

**Fig. 2:**
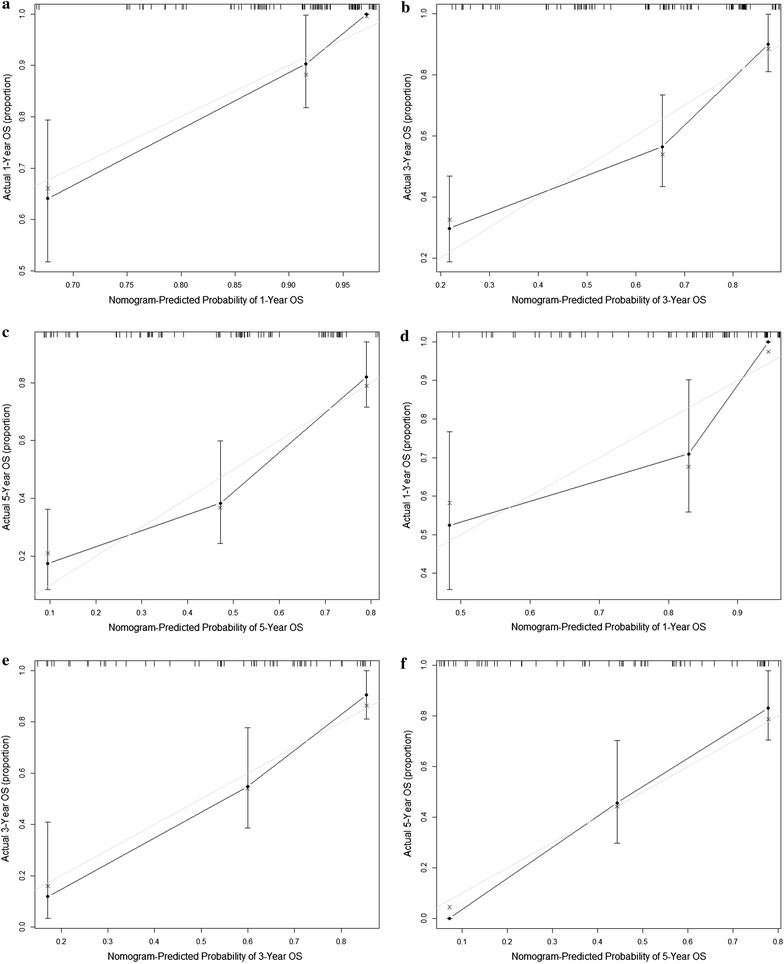
The calibration curves for predicting patient OS at (**a**) 1 year, (**b**) 3 years and (**c**) 5 years in the primary cohort and at (**d**) 1 year, (**e**) 3 years and (**f**) 5 years in the validation cohort. The nomogram model predicted OS is plotted on the x-axis, and the actual OS is plotted on the y-axis. Solid black line = performance of the nomogram model; closer alignment with the diagonal gray line represents a better estimation

The predictive accuracies for OS in NSCLC patients with chronic HBV infection between the nomogram model and conventional TNM staging systems were compared by calculating the Harrell’s C-index (Table 3). In the primary cohort, the C-index was 0.780 (95% confidence interval (CI) 0.733–0.827), which was significantly higher than that of the TNM staging system, with a value of 0.693 (95% CI 0.640–0.746, *P *< 0.01). This result was also confirmed in the validation cohort, where the C-index of the nomogram model (0.786, 95% CI 0.731–0.841) was higher than the C-index of the TNM staging system (0.704, 95% CI 0.642–0.766, *P* < 0.01). Calibration curves at 1, 3 and 5 years were then used to assess the agreement between the predicted and actual outcomes (Fig. 2). The diagonal gray line represents the actual OS probability, while the solid black line represents the performance of the nomogram model in predicting the OS probability. The two lines overlap closely, indicating that the nomogram model provided better estimations in the patient cohort.

**Table 3:** The C-index of nomogram model and TNM stage for prediction of OS in the primary cohort and validation cohort

Factor	Primary cohort		Validation cohort	
	C-index (95% CI)	*p*	C-index (95% CI)	*p*
Nomogram model	0.780 (0.733–0.827)		0.786 (0.731–0.841)	
TNM stage	0.693 (0.640–0.746)		0.704 (0.642–0.766)	
Nomogram model vs TNM stage		< 0.01		< 0.01

Nomogram Model: including eight risk factors (age, size, pTNM, treatment, APOAI, APOB GGT and LDH)

*C-index* concordance index, *CI* confidence interval

### Performance of the nomogram model in stratifying patient risk

**Fig. 3:**
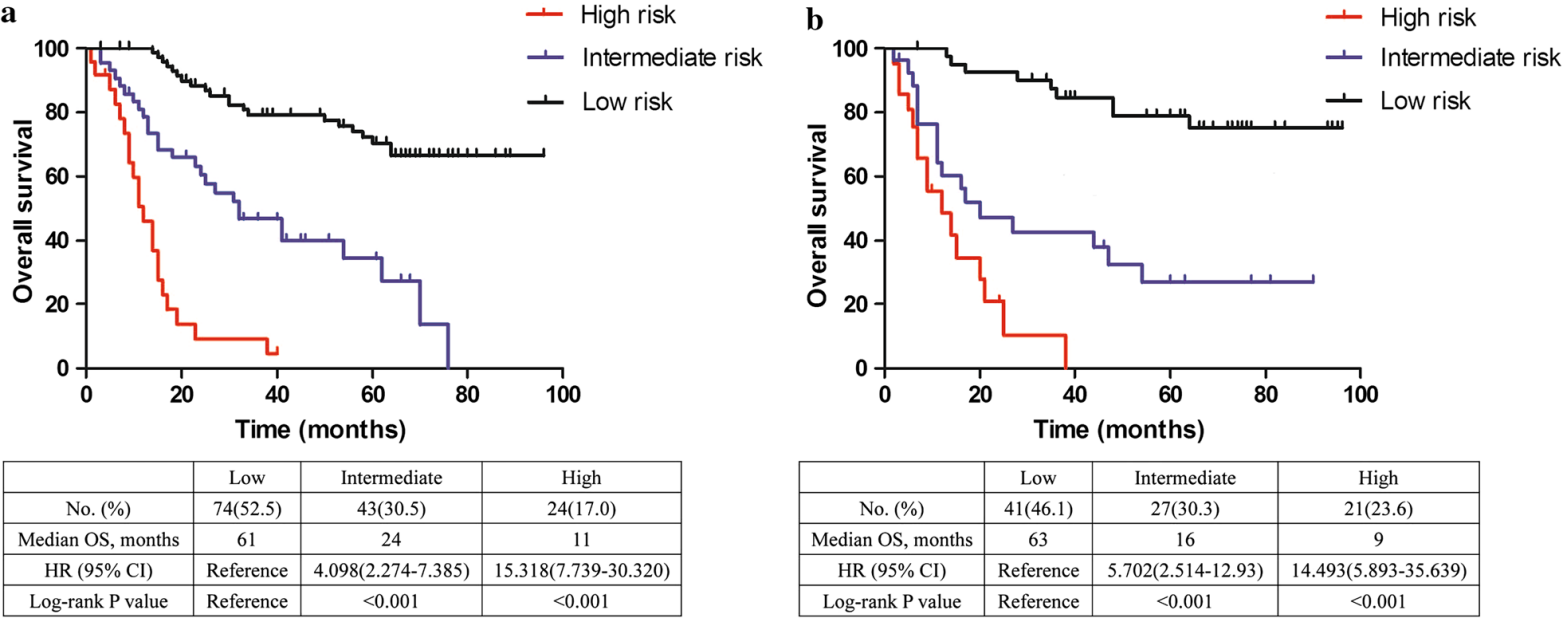
Graphs showing the results of the Kaplan–Meier curves for all three groups based on the prediction from the nomogram model in the primary cohort (left **a**) and in the validation dataset (right **b**). A significant association of the radiomics signature with the OS was shown in the training dataset, which was then confirmed in the validation dataset

Next, we determined the cutoff values using the X-tile program by grouping the patients in the primary cohort evenly into the following 3 groups based on the predictions of the nomogram model: low-risk prognosis (TPS ≤ 13.5, 74 patients), intermediate-risk prognosis (13.5 < TPS ≤ 20.0, 43 patients) and high-risk prognosis (TPS > 20.0, 24 patients). The low-risk group had the highest probability of survival (95.9% for 1 year, 68.9% for 3 years and 55.4% for 5 years), followed by the intermediate-risk group with survival probabilities of 76.7, 37.2 and 14.0% for 1, 3 and 5 years, respectively. The high-risk group had survival probabilities of 45.8, 8.3 and 0% for 1, 3 and 5 years, respectively. The differences in survival could be used to discriminate these three groups (Table 4). We then applied the cutoff values to plot Kaplan–Meier curves in the primary cohort and the validation cohort (Fig. 3). Both were significantly associated with OS outcomes within the three groups (*P *< 0.001).

**Table 4:** Cox regression analysis for groups based on the model in the primary cohorts

Groups	OS mean	1-year (%)	3-years (%)	5-years (%)	Sig.	HR	95% CI for HR	
							Lower	Upper
Low	75.976	95.9	68.9	55.4	–	–	–	–
Intermediate	38.839	76.7	37.2	14.0	< 0.001	4.098	2.274	7.385
High	13.852	45.8	8.3	0.0	< 0.001	15.318	7.739	30.320

Groups were divided by cutoff values of total prognostic scores (TPS) cumulated from nomogram we designed. (TPS ≤ 13.5, 13.5 < TPS ≤ 20.0, TPS > 20.0) (group lowest risk, intermediate risk and high risk with 74, 43 and 24 patients, respectively.). *CI* confidence interval, *HR* hazard ratio, *OS* overall survival

### Decision curve analysis for 5-year survival predictions

**Fig. 4:**
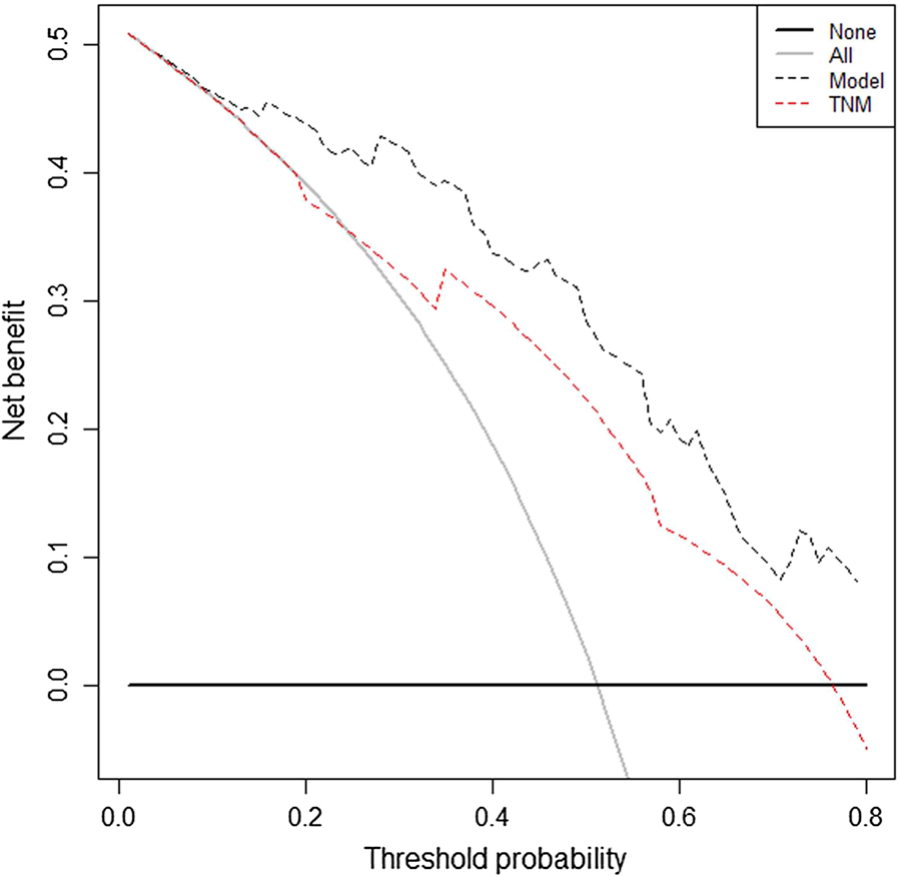
Decision curve analysis for the 5-year survival predictions. In the decision curve analysis, the y-axis indicates net benefit, calculated by summing the benefits (true positives) and subtracting the harms (false positives). The nomogram model (black dotted line) had the highest net benefit compared with the TNM staging system (red dotted line). The straight line represents the assumption that all the patients will die, and the horizontal line represents the assumption that none of the patients will die

Figure 4 presents the results of the decision curve analysis at 5 years. The results showed that our nomogram model had a higher overall net benefit than did the traditional TNM staging system across a wide range of threshold probabilities.

### Correlations among the variables of the nomogram model

**Fig. 5:**
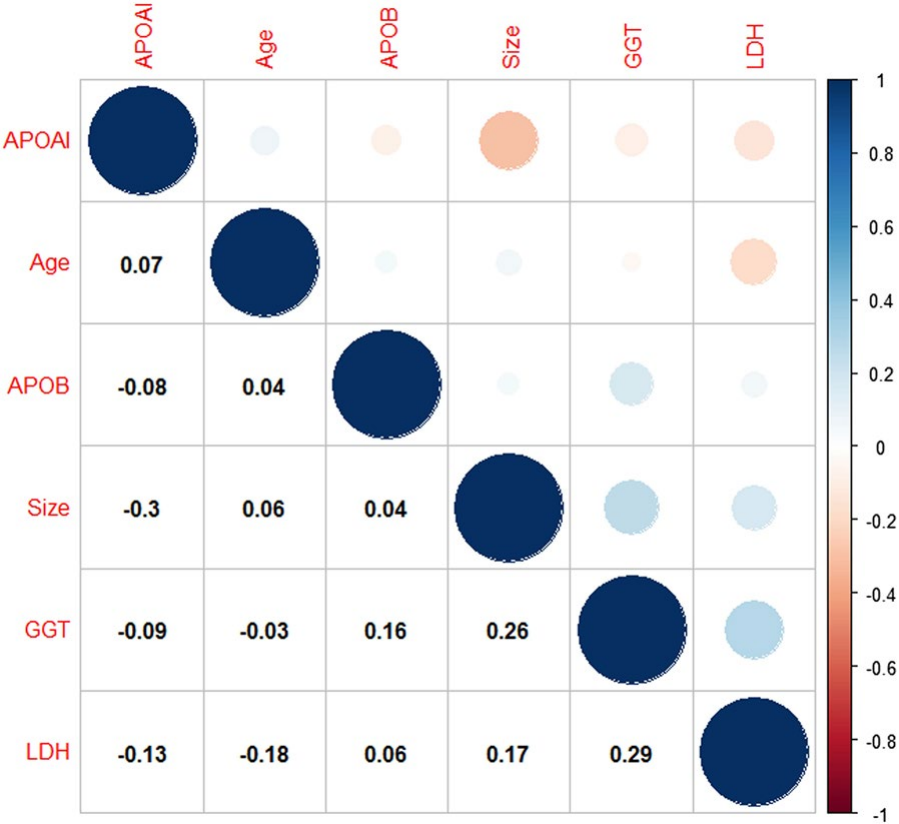
The correlations among the various variables of the nomogram model

Figure 5 shows the correlations among the different variables of the nomogram model. In this plot, positive correlations are displayed in blue and negative correlations in red. The color intensity and the size of the circle are proportional to the correlation coefficients. Additionally, the numbers in the graph show the Pearson’s correlation coefficient between the different variables. The results revealed that there was not a significant correlation among the various variables.

## Discussion

In this study, we evaluated the prognostic power of LFIs in NSCLC patients with HBV infection, taking advantage of the ability of a nomogram model that combined LFIs with clinicopathological characteristics to establish an effective predictive nomogram model for NSCLC patients with HBV infection. To our knowledge, this study is the first to establish a prognostic nomogram model for NSCLC patients with HBV infection based on the clinicopathologic data of 230 patients. The model included age, TNM stage, tumor size, treatment, APOAI, APOB, GGT, and LDH. Our nomogram model had better discriminatory ability than the current AJCC TNM classification system. The nomogram model also had a higher overall net benefit than the TNM staging system at 5 years.

Many studies have shown that LFIs are correlated with cancer prognosis. Our model included APOAI, APOB, GGT and LDH, which are good prognostic markers in different types of cancers. APOAI has been shown to have cardioprotective, anti-inflammatory, anti-viral, anti-parasitic, anti-bacterial and anti-tumor activity functions [20]. APOAI is a useful prognostic factor in breast cancer [21], renal cell carcinoma [22], nasopharyngeal carcinoma [23] and lung cancer [24]. APOB is a major structural protein for atherogenic APOB-containing lipoproteins [25]. The levels of APOB are positively associated with the risk of colorectal cancer, breast cancer, lung cancer [26, 27]. In addition, our study is the first to report that APOB is correlated with lung cancer prognosis. GGT is a membrane-bound enzyme involved in the metabolism of glutathione. Several previous studies revealed that GGT is related to tumor development, progression, invasion, drug resistance and prognosis [28, 29]. Elevated serum levels of GGT were also found to be associated with poorer prognosis in several human cancers. LDH, a hypoxia regulator, plays a vital role in alternative metabolic pathways of cancer cells [30]. Serum LDH levels could be a low-cost and useful prognostic factor in patients with lung cancer [31, 32].

Compared with previous studies, our results differed slightly in that our model excluded ALP and ALB. Serum ALP is used as an indicator of hepatic and bone diseases as it is convenient to measure. Arife et al. [7] showed that the risk of progression with normal levels of ALP was significantly higher than the risk with high ALP levels among advanced NSCLC patients. ALP is an independent prognostic factor related to OS and progression-free survival in NSCLC patients. Serum ALB is used to assess nutritional status. Many studies have shown that serum albumin as an independent prognosticator of survival in lung cancer [33, 34]. These results may be explained by the different prognostic outcomes between NSCLC patients with HBV infection and those of patients without HBV infection. Furthermore, the cutoff value of ALB was different from that of previous reports. Here, we adopted the X-tile program to choose the optimal cutoff points, which may have led to the different results.

HBV is a noncytopathic virus that does not cause direct damage to liver cells. Instead, it is the immune system’s aggressive response to the virus that leads to inflammation and damage to the liver [35, 36]. Thereby, inflammation and biochemical indicators of liver function are correlated with cancer prognosis and influence the prognosis of cancer patients. Therefore, we developed an effective nomogram model to predict OS in NSCLC patients with HBV infection.

In addition to these strengths, our study has various limitations. First, due to the retrospective nature of our study, we cannot avoid potential biases, and we enrolled relatively few patients. Second, the data were obtained from a single center and represent a small sample size. Therefore, further multi-center studies using higher sample sizes are needed to externally validate the nomogram model to verify whether our findings are universally applicable. Third, we only analyzed the impact of the biochemical indicators of liver function on the prognosis of NSCLC patients with HBV infection. Other prognostic factors such as inflammatory factors, HBV DNA, serum carcinoembryonic antigen (CEA) [37] and oncogenic mutations [38] were not included. These factors should be considered in future studies. Despite these limitations, the nomogram model was effective and may be useful in predicting the outcomes of NSCLC patients with HBV infection.

## Conclusion

In summary, we developed and validated an effective nomogram model predicting the 1-, 3- and 5-year OS in NSCLC patients with HBV infection. The proposed nomogram in this study showed a better level of discrimination and accuracy than the current AJCC TNM classification system. Furthermore, it could be useful for patient counseling and individualized prediction of survival for NSCLC patients with HBV infection.
